# Diagnostic accuracy of high *b*-value diffusion weighted imaging for patients with prostate cancer: a diagnostic comprehensive analysis

**DOI:** 10.18632/aging.203164

**Published:** 2021-06-22

**Authors:** Chao Li, Na Li, Zhanzhan Li, Liangfang Shen

**Affiliations:** 1 Department of Oncology, Xiangya Hospital, Central South University, Changsha, Hunan Province 410008, China

**Keywords:** prostate cancer, high *b*-value, diffusion-weighted imaging, diagnostic accuracy, meta-analysis

## Abstract

We performed a meta-analysis to assess the diagnostic accuracy of high *b*-value diffusion-weighted imaging for patients with prostate cancer. A comprehensive literature search of the PubMed, Excerpta Medica Database, Cochrane Library, China National Knowledge Infrastructure, China Biology Medicine disc, and Wanfang databases from January 1, 1995, to April 30, 2021, was conducted. The quality of the retrieved papers was assessed using the Quality Assessment of Diagnostic Accuracy Studies 2. The sensitivity, specificity, positive likelihood ratio, negative likelihood ratio, diagnostic odds ratio, and their 95% confidence intervals (CIs) were evaluated using bivariate mixed effects models. A total of twenty-four articles matched the selection criteria and were finally included after screening the titles, abstracts, and full texts of 641 initial articles. The pooled sensitivity and specificity (95% CI) were 0.84 (0.80–0.87) and 0.87 (0.81–0.91), respectively. The pooled positive and negative likelihood ratios (95% CI) were 6.4 (4.4–9.3) and 0.19 (0.16–0.23), respectively. The diagnostic odds ratio was 34 (95% CI: 22–51). The area under the summary receiver operator characteristic curve was 0.91 (95% CI: 0.88–0.93). Subgroup analysis presents similar results. The diagnostic accuracy of high *b*-value diffusion-weighted imaging was similarly high in the qualitative and quantitative evaluation of prostate cancer.

## INTRODUCTION

Prostate cancer is the second most commonly diagnosed cancer and the sixth leading cause of cancer-related death in men [1]. Early diagnosis and treatment are of great importance. Serum prostate-specific antigen (PSA) detection is the primary option for screening prostate cancer [2]. However, PSA is specific to prostate tissue rather than tumor tissue, and prostatitis, urinary tract infection and even prostate massage can lead to an increase in PSA levels. It has been reported that the specificity of PSA is very low when 4.0 ng/ml serum PSA is used as a threshold [3]. PSA detection alone may cause a high false-positive rate and lead to a large number of unnecessary biopsies [4]. Therefore, PSA-based screening for prostate cancer is controversial.

In clinical practice, magnetic resonance imaging (MRI) has been widely used to detect prostate cancer. It differs from other techniques such as computed tomography and ultrasound since it produces excellent soft tissue contrast without harmful ionizing radiation; MRI also provides imaging evidence for the clinical examination of prostate cancer location, staging, postoperative follow-up, and the evaluation of tumor invasion [5]. MRI mainly included five imaging parameters: T1-weighted imaging, T2-weighted imaging, diffusion-weighted imaging (DWI), magnetic resonance spectroscopy, and dynamic contrast-enhanced imaging. DWI can estimate morphological changes in prostate tissue that occur with the induction of plasticity by probing water diffusion and can qualitatively and quantitatively evaluate the cellular and histological structure of prostate cancer [6]. The *b*-value is one of the primary parameters influencing DWI results. According to the Prostate Imaging Reporting and Data System version 2, high *b*-values (1,400–2,000 s/mm^2^) are favored over standard *b*-values (800–1,000 s/mm^2^) for improving tumor detection since they can qualitatively distinguish lesions and normal prostate tissue. The latter shows high signal intensity on DWI, which may not be suppressed even at a *b*-value of 1,000 s/mm^2^, resulting in obscured prostate cancer [7, 8]. Higher *b*-value DWI has been continuously applied in clinical practice. Although a high *b*-value reduces the signal-to-noise ratio of images and may distort images, it can reduce the T2 penetration effect and microcirculation perfusion of images and more truly reflect the tissue and cytological structure. Because of conflicting results from qualitative and quantitative studies, it is not definitively known whether high *b*-value DWI improves the diagnostic accuracy of prostate cancer. The purpose of this meta-analysis was therefore to assess the diagnostic performance of high *b*-value DWI for detecting prostate cancer.

## RESULTS

### Search process and general characteristics

**Figure 1:**
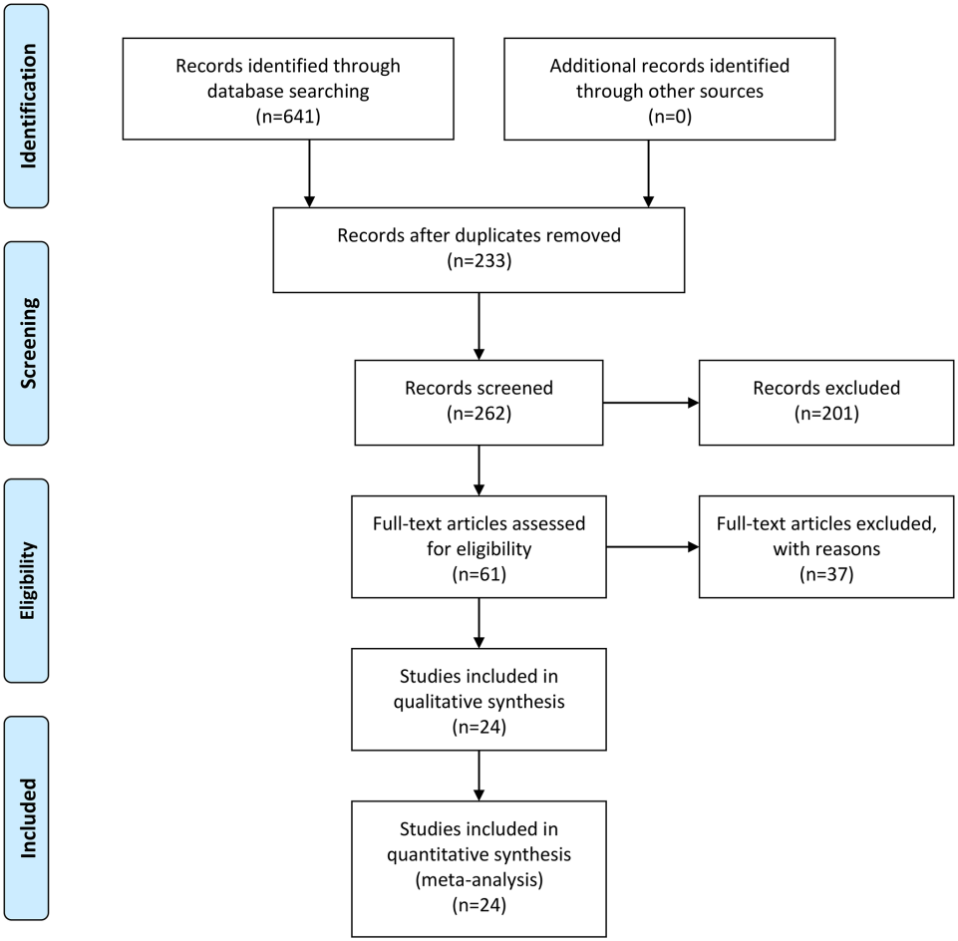
Flow diagram of studies selection process.

A flow chart of the study selection process is shown in Figure 1. A total of 641 articles were retrieved during the original search of publications from January 1, 1995, to December 31, 2020, and 233 remained after excluding duplicates among the different databases during the second search round. After excluding reviews, comments, case reports, and studies unrelated to our topics, 61 articles were reviewed. Twenty-four articles remained after omitting articles that did not mention diagnostic accuracy or that had insufficient data [9–32], [13–36]. The excluded studies with reasons are provided in Supplementary Material 1. The backgrounds and designs of these studies are shown in Table 1 and Table 2. Thirteen studies selected populations with PCa, and two studies were performed on suspected cases. The methods for identifying *b*-values were different: ten studies used motion-probing gradients, and five studies used signal extrapolation by fitting models. All DWI scans were acquired using single-shot spin-echo echo-planar imaging. The biopsy types included targeted biopsy, prostatectomy and systematic biopsy. The used tissue amounts were hardly reported in the studies. The primary characteristics of the selected studies are shown in Table 3. Among the enrolled studies, 17 were prospective studies and 7 were retrospective studies. Among the studies, a total of 1887 patients with 11374 lesions were analyzed. The mean age of all patients was 66.3 years. The MRI field intensity used in most of the studies was 3.0 T, with only three studies using a field intensity of 1.5 T. An endorectal coil was used in only two study. Table 1 also lists the MRI suppliers, gold standards, and DWI diagnostic measures. Table 2 shows that each study contained a *b*-value of 2,000 s/mm^2^, and the highest *b*-value was 4,500 s/mm^2^. The ranges of the sensitivities and specificities in all studies were 44.0–98.6% and 50.0–99.4%, respectively.

**Table 1:** Background and design information of included studies in the meta–analysis.

**Author**	**Country**	**Study design**	**Patients**	**Mean age (y)**	***b*-value**	**PSA(μg/L)**	**Field Intensity(T)**	**MRI Supplier**	**Endorectal Coil**	**DWI diagnostic measure**
Adubeio	Portugal	prospective	43	63	0,50,100,150,200,500,800,1100,1400, 1700,2000	–	3.0	Philips	No	Visual evaluation
Barral	France	prospective	35	64	0.2000	–	3.0	Siemens	No	Visual evaluation
Wang	China	prospective	67	68.3	0,800,1500,2000	6.50~530	3.0	Philips	No	Visual evaluation
Li	China	prospective	47	68	0,400,1000,1500,2000	–	3.0	Siemens	No	Visual evaluation
Koo	Korea	retrospective	80	66	0,300,700,1000,2000	1.24~56.98	3.0	Philips	No	Visual evaluation
Feng	China	prospective	56	66.71	0,1000,2000,3200, 4500	2.06~1000	3.0	GE	No	ADC value
Kim	Korea	retrospective	48	66	0,1000,2000	2.30~23.2	3.0	Philips	No	Visual evaluation
Wang	China	prospective	80	66	0,300,700,1000,2000	1.24~56.98	3.0	GE	No	Visual evaluation
Zhang	China	prospective	40	67	0,1000,2000,3000	–	3.0	GE	No	Visual evaluation
Peng	USA	retrospective	48	62.5	0,50,200,1000,1500, 2000	0.80~256	1.5	Philips	Yes	ADC
Meng	China	prospective	80	72.45	0,1000,2000	–	3.0	GE	No	ADC
Zhang	China	retrospective	170	59.5	0,600,800,1000,1500. 2000,2500,3000	–	1.5	GE	No	Visual evaluation
Ning	China	retrospective	97	64	0,1200,2000	0.60~63	3.0	GE	No	Visual evaluation
Wang	China	prospective	60	85.5	0,2000	4.10~150.2	3.0	Siemens	No	Visual evaluation
Xue	China	prospective	37	62	0,500,1000,1500,2000	3.06~153	3.0	Philips	No	ADC
Costa	USA	Prospective	49	61	0.2000	0.9–26	3.0	-	both	Visual evaluation
Katahira et al.	Japan	retrospective	201	69	0,1000.2000	2.6–114	1.5	Philips	No	Visual evaluation
Ohgiya	Japan	retrospective	73	70	0,500.1000,2000	11.7	3	Siemens	No	Visual evaluation
Rosenkrantz	USA	retrospective	106	62	50,1000,2000	4.5–130	3	Siemens	No	Visual evaluation
Stanzione	Italy	Prospective	82	65	0,400,2000	8.8	3	Siemens	No	Visual evaluation
Thestrup	Denmark	retrospective	204	64.1	0,1000,2000	2.2–120	3	Philips	No	Visual evaluation
Ueno1	Japan	retrospective	73	67	0,1000,2000	2.9–49	3	Philips	No	Visual evaluation
Ueno2	Japan	retrospective	80	67	0,1000,2000	2.9–49	3	Philips	No	Visual evaluation
Ueno	Japan	retrospective	31	65	0,2000	4.7–16.5	3	Philips	No	Visual evaluation

Abbreviations: PSA: prostate-specific antigen; MRI: magnetic resonance imaging; DWI: diffusion-weighted imaging; TRUS: transrectal ultrasound; TSB: tissue sample biopsy; RP: radical prostatectomy; ADC: apparent dispersion coefficient.

**Table 2:** Technology characteristics of included study.

**Author**	**Population**	**Biopsy type**	**Type of sequences**	**TR/TE (ms)**	**Diffusion times**	**Methods for identifying**
Adubeio	PCa	Targeted/prostatectomy	SS-SE-EPI	3258/66	13:21 min	signal extrapolation
Barral	PCa	Partly glands after prostatectomy	SS-SE-EPI	5200/70	2 min 15 s	motion probing gradients
Wang	PCa	Targeted biopsy	SS-SE-EPI	4500/93	N/A	motion probing gradients
Li	PCa	prostatectomy	SS-SE-EPI	5300/84	N/A	motion probing gradients
Koo	PCa	prostatectomy	SS-SE-EPI	4830–4840/75–76	Less than 5 min	motion-probing gradients
Feng	PCa	Targeted biopsy	SS-SE-EPI	2500/84.1	10 min 20 s	signal extrapolation
Kim	PCa	prostatectomy	SS-SE-EPI	2924–2950/93–95	3 min 20 s	signal extrapolation
Wang	PCa	prostatectomy	SS-SE-EPI	4830–4840/75–76	N/A	motion probing gradients
Zhang	PCa	systematic biopsy	SS-SE-EPI	5000/73	N/A	signal extrapolation
Peng	PCa	prostatectomy	SS-SE-EPI	2948–8616/71–85	N/A	signal extrapolation
Meng	excessive nocturnal urination and dysuria	systematic biopsy	SS-SE-EP	3000/55	N/A	motion probing gradients
Zhang	PCa	systematic biopsy	SS-SE-EP	7000/83.7	N/A	motion probing gradients
Ning	PCa	Targeted biopsy	SS-SE-EPI	2000/54	N/A	motion probing gradients
Wang	elevated PSA	systematic biopsy		2000/58	N/A	motion probing gradients
Xue	PCa	systematic biopsy	SS-SE-EPI	6000/90	23, 3:54, 10:12	motion probing gradients
Costa	PCa	prostatectomy	SS-SE-EPI	3938/110	4 min 8 s	both
Katahira et al.	PCa	prostatectomy	SS-SE-EPI	5260/56	1 min 54 s	motion probing gradients
Ohgiya	PCa	prostatectomy	SS-SE-EPI	3200/80	4 min	motion probing gradients
Rosenkrantz	PCa	prostatectomy	SS-SE-EPI	3500/81	5 min 5 s	motion probing gradients
Stanzione	PCa	prostatectomy	SS-SE-EPI	4900/89	6 min 28	motion probing gradients
Thestrup	PCa	Partly prostatectomy	SS-SE-EPI	9867/71	6 min 33 s	motion probing gradients
Ueno1	PCa	prostatectomy	SS-SE-EPI	4000/65	3 min 20 s	motion probing gradients
Ueno2	PCa	prostatectomy	SS-SE-EPI	4000/65	3 min 20 s	motion probing gradients
Ueno	PCa	prostatectomy	SS-SE-EPI	4000/65	3 min 20 s	motion probing gradients

Abbreviations: ADC: apparent diffusion coefficient; SS-SE-EPI: single-shot spin-echo echo-planar imaging.

**Table 3:** General characteristics of included studies in the meta-analysis.

**Study**	**Year**	**Lesions**	**TP**	**FP**	**FN**	**TN**	**Sensitivity (%)**	**Specificity (%)**	***b*-value (s/mm^2^)**
Adubeio et al.	2018	76	40	3	3	30	93.2	90.9	2000
Barral et al.	2015	113	62	1	16	34	79.1	99.4	2000
Wang et al.	2017	93	40	5	13	35	75.4	87.5	2000
Li et al.	2015	47	23	2	4	18	85.2	90	2000
Koo et al.	2013	800	152	22	53	573	74	96	2000
Feng et al. [1]	2017	336	136	51	2	147	98.6	74.2	2000
Feng et al. [2]	2017	336	124	28	14	170	89.9	85.9	3200
Feng et al. [3]	2017	336	117	15	21	183	84.8	92.4	4500
Kim et al.	2010	672	128	40	52	452	71	92	2000
Wang et al.	2015	136	81	12	29	292	74	96	2000
Zhang et al. [1]	2016	40	19	5	3	13	86.4	72.2	2000
Zhang et al. [2]	2016	40	20	3	2	15	90.9	83.3	3000
Peng et al.	2013	104	49	6	12	37	80	86	2000
Meng et al.	2017	80	38	3	5	34	88.37	91.89	2000
Zhang et al. [1]	2017	170	124	7	19	20	86.7	78.6	2000
Zhang et al. [2]	2017	170	133	6	10	21	93.0	76.9	2500
Zhang et al. [3]	2017	170	118	7	25	20	82.6	73.4	3000
Ning et al.	2018	138	50	6	11	71	82	92.2	2000
Wang et al.	2016	60	32	2	4	22	88.9	91.7	2000
Xue et al.	2017	52	19	2	8	23	70.9	89.1	2000
Costa et al.	2016	118	20	19	6	73	44.0	79.0	2000
Katahira et al.	2011	4815	1162	332	425	2896	73.0	90.0	2000
Ohgiya et al.	2012	73	42	2	13	16	76.0	89.0	2000
Rosenkrantz et al.	2015	636	46	10	16	564	74.0	98.0	2000
Stanzione et al.	2016	87	29	1	5	52	85.0	98.0	2000
Thestrup et al.	2016	204	65	116	3	20	96.0	15.0	2000
Ueno1 et al.	2013	584	276	79	65	164	81.0	68.0	2000
Ueno2 et al.	2013	640	272	95	55	218	83.0	50.0	2000
Ueno et al.	2015	248	86	51	35	76	71.0	60.0	2000

Abbreviations: TP: true positive; FP: false positive; FN: false negative; TN: true negative.

### Quality assessment

**Figure 2:**
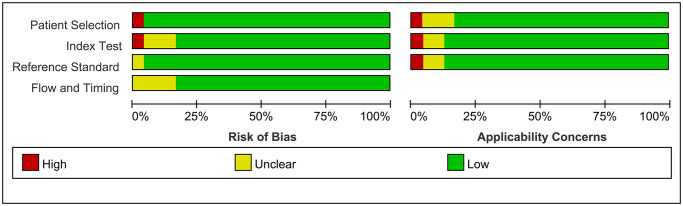
Risk of Bias and applicability concerns graph: Judgments about each domain presented as percentages across included studies.

**Figure 3:**
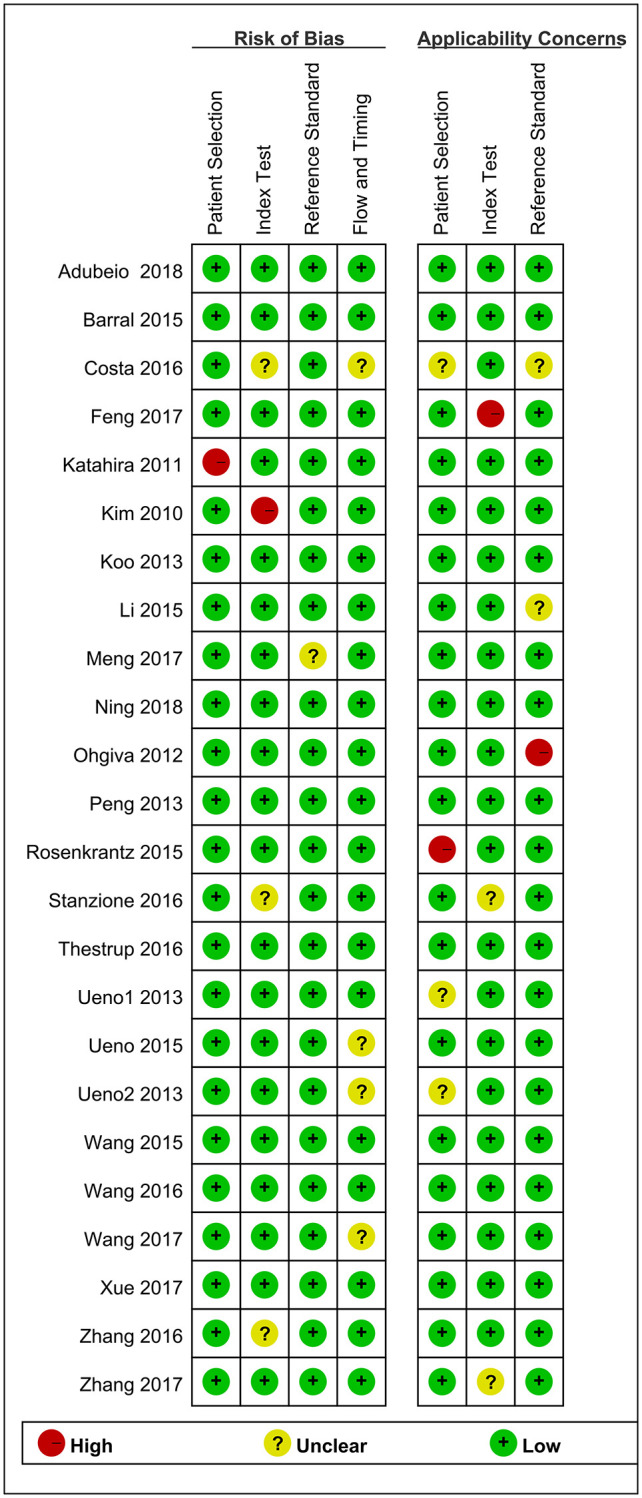
Risk of Bias and applicability concerns summary: judgments about each domain for each included study.

As shown in Figure 2 and Figure 3, the risk of bias consisted of flow and timing, patient selection, index test, and reference standard, whereas applicability concerns consisted of the last three domains but not flow and timing. Only five study had a high risk, and one was unclear in terms of the index test for both the risk of bias and applicability concerns. For the reference standard, one studies were unclear, and four study were unclear in terms of flow and timing, three studies were unclear in index test. Overall, the quality of the identified studies was high.

### Pooling results

**Figure 4:**
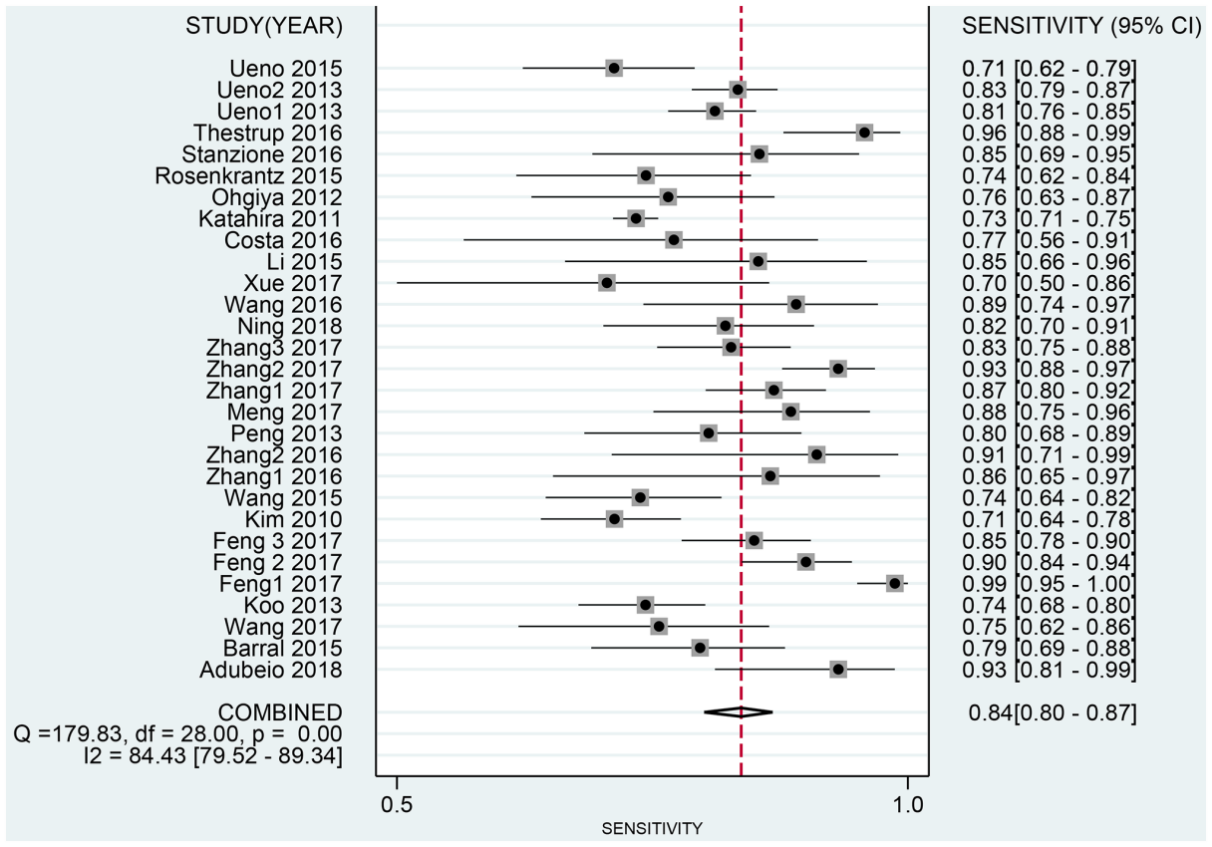
Forest plot of pooled sensitivity of diagnostic accuracy of high *b*-value DWI for detecting prostate cancer.

**Figure 5:**
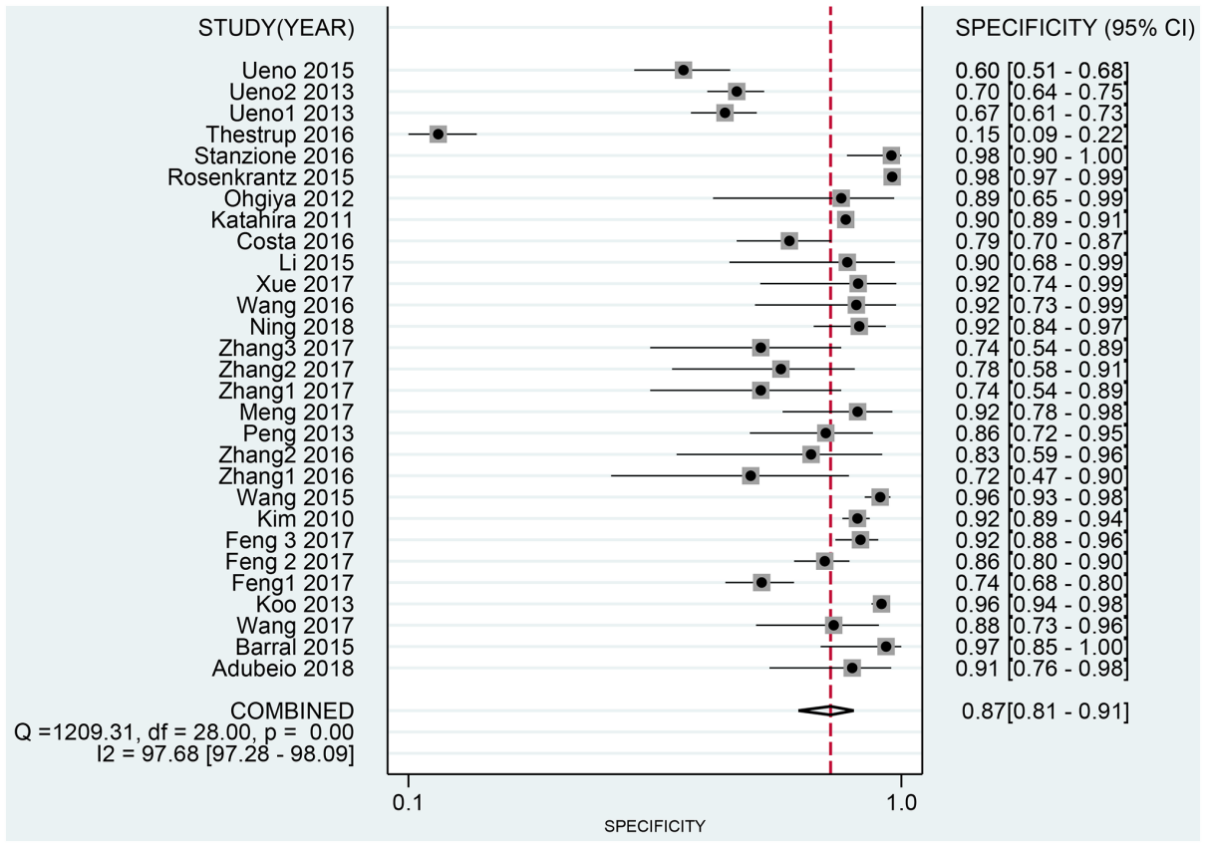
Forest plot of pooled specificity of diagnostic accuracy of high *b*-value DWI for detecting prostate cancer.

**Figure 6:**
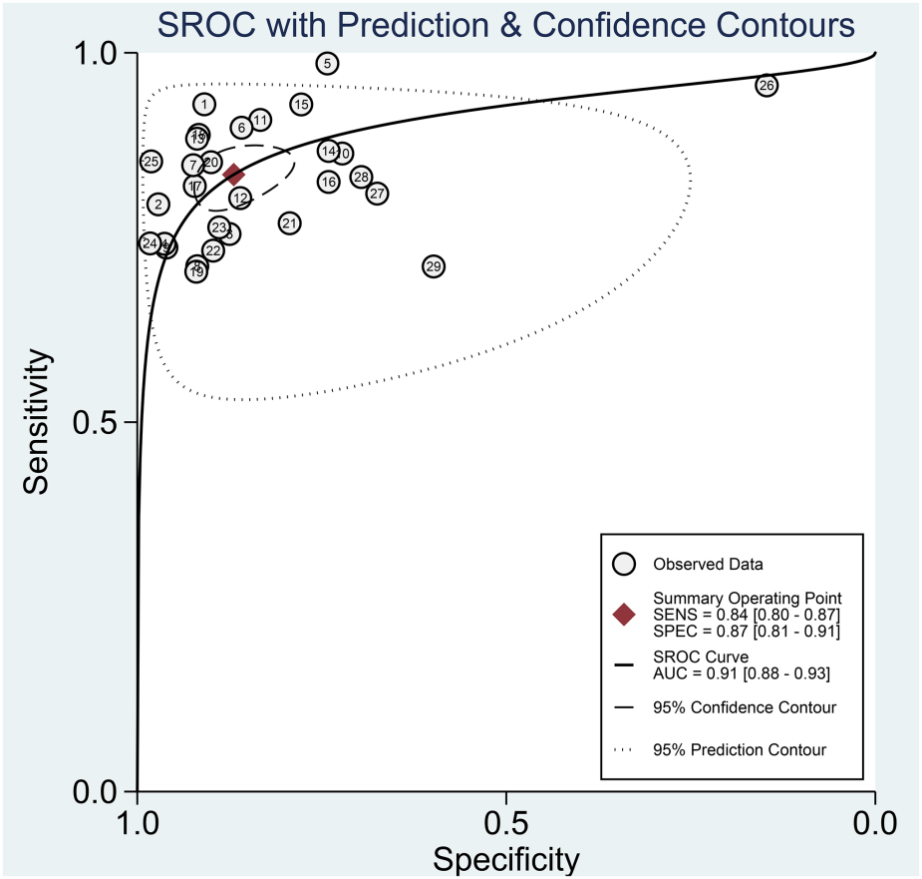
The SROC curve of diagnostic accuracy of high *b*-value DWI for detecting prostate cancer.

**Figure 7:**
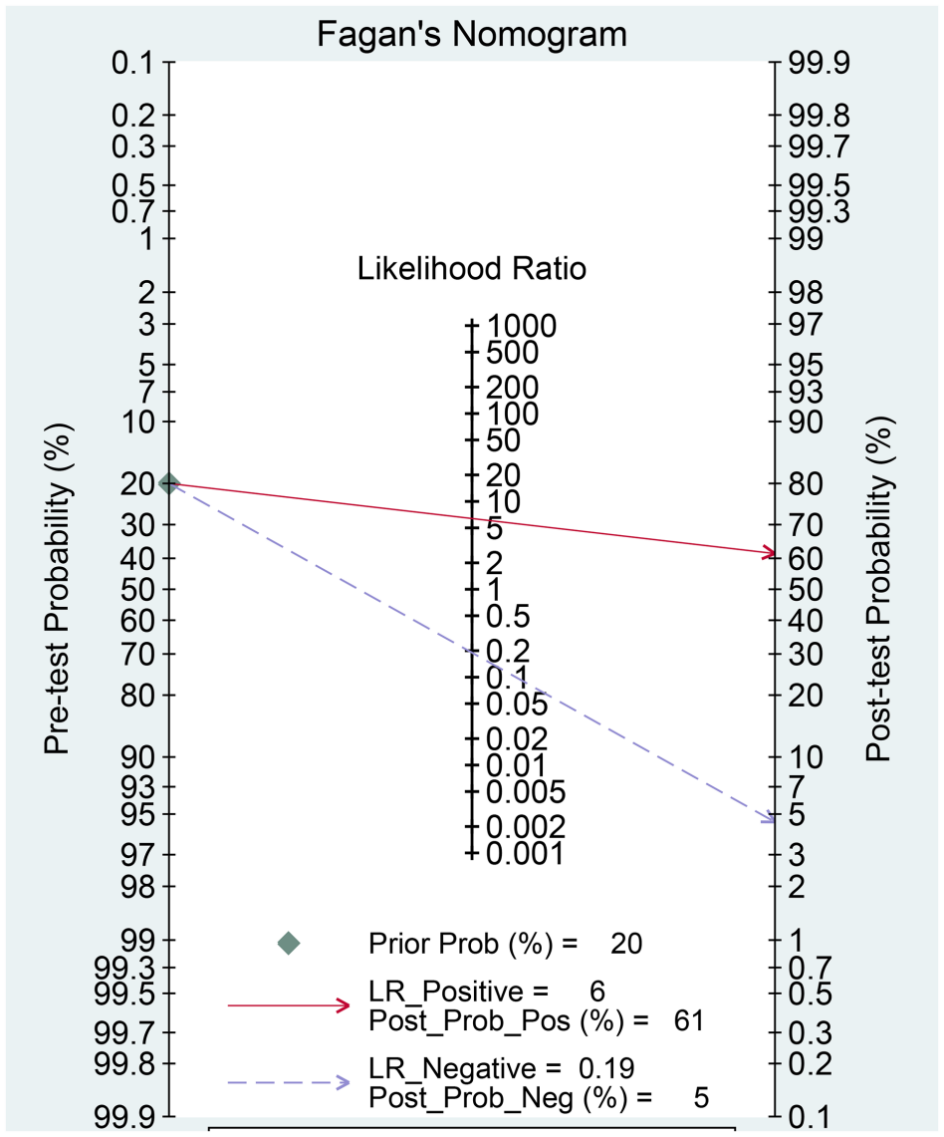
Fagan diagram evaluating the overall diagnostic value of high *b*-value for detecting prostate cancer.

**Figure 8:**
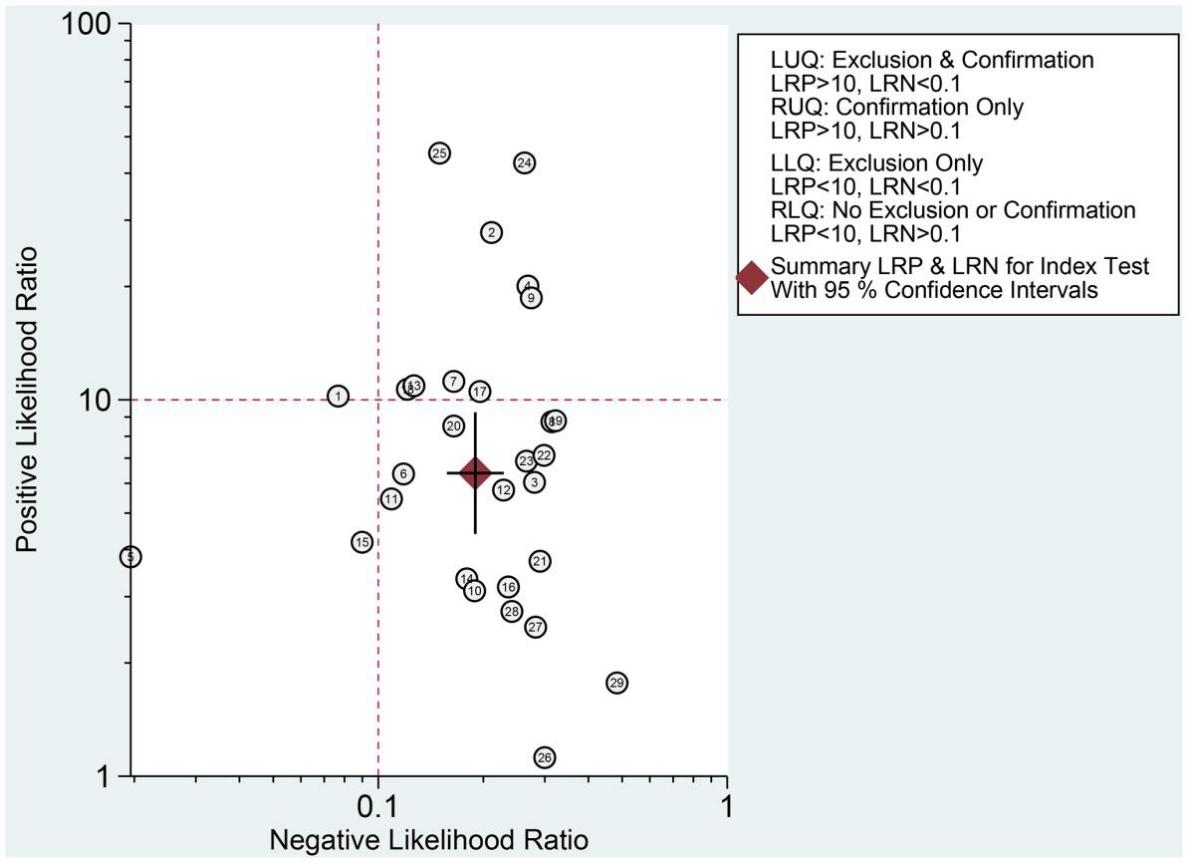
Likelihood ratio scatter gram.

The Spearman correlation indicated no threshold effect (*r* = 0.317, *P* = 0.094). From the data obtained, we determined pooled sensitivity and specificity values of 0.84 (95% CI: 0.80–0.87) and 0.87 (95% CI: 0.81–0.91), respectively (Figure 4 and Figure 5). The AUC was 0.941(0.88–0.93) (Figure 6). The PLR and NLR were 6.4 (95% CI: 4.4–9.3, Supplementary Materials 2 and 3) and 0.19 (95% CI: 0.16–0.23), respectively, and the DOR was 34 (95% CI: 22 to 51, Supplementary Material 4). According to Fagan's nomogram (Figure 7), when the pretest probability was 20%, the corresponding post-test probability was 61% using the PLR and 5% using the NLR. The diagnostic performance was visualized by a likelihood ratio scattergram (Figure 8). All of these results suggest that the degree of diagnostic accuracy of high *b*-value DWI for detecting prostate cancer was relatively high.

### Subgroup analysis

We conducted subgroup analyses of six subgroups (study design, number of patients, mean age of the patients, MRI field intensity, *b*-value, and DWI diagnostic measures) to identify the sources of heterogeneity. All results are shown in Table 4. The study design was divided into prospective and retrospective studies, and the sensitivity, specificity, PLR, NLR, and DOR showed no significant differences, but the AUC was significantly different, suggesting that it was a cause of the heterogeneity. The sensitivity, specificity, PLR, DOR, and AUC values were slightly superior for > 50 patients than for ≤ 50 patients, and the same trend was observed between those aged > 65 years and those aged ≤ 65 years. However, neither the number of patients nor the mean age showed a significant difference, suggesting that they did not contribute to the heterogeneity. MRI field intensity was divided into 1.5 T and 3.0 T, and the integrated sensitivity, specificity, PLR, NLR, DOR, and AUC values were 0.84, 0.83, 4.9, 0.19, 25, and 0.90 for 1.5T and 0.84, 0.88, 7.0, 0.19, 37, and 0.91 for 3.0T, respectively. There were no significant differences in any of these values between the two field intensities, suggesting that MRI field intensity did not contribute to the heterogeneity. Regarding *b*-values, the sensitivity, specificity, PLR, NLR, DOR, and AUC values were 0.83, 0.87, 6.6, 0.20, 33, and 0.90 for high *b*-values and 0.88, 0.86, 6.2, 0.14, 45, and 0.93 for ultrahigh *b*-values, respectively. Regarding DWI diagnostic measures, the sensitivity, specificity, PLR, NLR, DOR, and AUC values for ADC (quantitative) values were 0.89, 0.88, 7.6, 0.13, 58, and 0.94, and those for visual evaluation (qualitative) were 0.82, 0.86, 6.0, 0.21, 29, and 0.88. The results showed no significant differences between groups. Similar results were also found among different biopsy type, methods for identifying *b*-value. However, MRI supplier from Siemens and Ge seems to be prior to Philips (Philips: AUC (0.85, 95% CI: 0.82–0.88); Siemens: AUC (0.90, 95% CI: 0.88–0.83); Ge: AUC (0.94, 95% CI (0.91–0.96)) (Table 4). The heterogeneity was high within studies. The meta-regression indicated that publication year and population setting may cause the heterogeneity within studies (Table 5).

**Table 4:** Summary of pooled results.

**Subgroup**	**Sensitivity (95% CI)**	**Specificity (95% CI)**	**PLR (95% CI)**	**NLR (95% CI)**	**DOR (95% CI)**	**AUC (95% CI)**
**All studies**	0.84 (0.80–0.87)	0.87 (0.81–0.91)	6.4 (4.4–9.3)	0.19 (0.16–0.23)	34 (22–51)	0.91 (0.88–0.93)
**Study design**						
Prospective	0.87 (0.82–0.90)	0.90 (0.86–0.93)	8.6 (6.4–11.6)	0.15 (0.11–0.20)	58 (44–78)	0.95 (0.92–0.96)
Retrospective	0.81 (0.76–0.85)	0.83 (0.69–0.91)	4.6 (2.6–8.2)	0.23 (0.19–0.28)	20 (11–36)	0.86 (0.83–0.89)
**Patients**						
≤ 50	0.79 (0.73–0.84)	0.86 (0.79–0.92)	5.8 (3.6–9.5)	0.23 (0.18–0.32)	24 (12–48)	0.87 (0.84–0.90)
> 50	0.85 (0.80–0.88)	0.87 (0.79–0.93)	6.6 (4.0–10.8)	0.18 (0.14–0.22)	37 (23–61)	0.91 (0.88–0.93)
**Mean age**						
≤ 65	0.84 (0.80–0.88)	0.87 (0.80–0.92)	6.6 (4.3–10.1)	0.18 (0.14–0.23)	37 (23–60)	0.92 (0.89–0.94)
> 65	0.83 (0.78–0.88)	0.88 (0.83–0.92)	6.8 (4.8–9.7)	0.19 (0.15–0.25)	36 (24–55)	0.92 (0.89–0.94)
**Field intensity**						
1.5T	0.84 (0.77–0.89)	0.83 (0.75–0.89)	4.9 (3.3–7.1)	0.19 (0.14–0.27)	25 (17–38)	0.90 (0.87–0.93)
3.0T	0.84 (0.79–0.87)	0.88 (0.81–0.93)	7.0 (4.4–10.9)	0.19 (0.15–0.23)	37 (23–61)	0.91 (0.88–0.93)
***B*–value**						
High	0.83 (0.78–0.86)	0.87 (0.80–0.92)	6.6 (4.1–10.5)	0.20 (0.16–0.24)	33 (20–55)	0.90 (0.87–0.92)
Ultra–high	0.88 (0.84–0.92)	0.86 (0.78–0.91)	6.2 (4.0–9.5)	0.14 (0.10–0.19)	45 (26–78)	0.93 (0.91–0.95)
**DWI diagnostic measure**						
ADC value	0.89 (0.79–0.94)	0.88 (0.82–0.92)	7.6 (5.3–10.9)	0.13 (0.07–0.24)	58 (36–94)	0.94 (0.92–0.96)
Visual evaluation	0.82 (0.78–0.85)	0.86 (0.78–0.92)	6.0 (3.7–9.7)	0.21 (0.18–0.25)	29 (17–49)	0.88 (0.75–0.91)
**Population setting**						
PCa	0.84 (0.80–0.87)	0.86 (0.80–0.91)	6.2 (4.1–9.3)	0.19 (0.16–0.23)	33 (21–51)	0.90 (0.87–0.93)
**Biopsy type**						
Prostatectomy	0.79 (0.75–0.83)	0.87 (0.76–0.94)	6.2 (3.3–11.7)	0.24 (0.20–0.28)	26 (13–51)	0.85 (0.82–0.88)
Systematic biopsy	0.87 (0.82–0.90)	0.83 (0.76–0.88)	5.1 (3.6–7.2)	0.16 (0.12–0.22)	32 (19–53)	0.92 (0.89–0.94)
Targeted biopsy	0.89 (0.78–0.95)	0.88 (0.81–0.93)	7.5 (5.1–11.0)	0.12 (0.06–0.25)	61 (36–101)	0.94 (0.92–0.96)
**Methods for identifying *b*–value**						
Signal extrapolation	0.89 (0.81–0.94)	0.87 (0.82–0.90)	6.8 (5.2–8.7)	0.12 (0.07–0.21)	55 (35–87)	0.93 (0.91–0.95)
Motion probing gradients	0.82 (0.78–0.85)	0.87 (0.78–0.93)	6.3 (3.7–10.7)	0.21 (0.18–0.25)	30 (17–53)	0.88 (0.84–0.90)

**Table 5:** Meta-regression for heterogeneity within studies.

**Parameter**	**Estimate (95% CI)**	***P***
Year of publication	81.41 (60.32–100.00)	0.00
Age	0.00 (0.00–100.00)	0.44
Sample size	0.00 (0.00–100.00)	0.71
Field intensity	0.00 (0.00–100.00)	0.57
DWI diagnostic measure	0.00 (0.00–100.00)	0.82
Population setting	62.30 (15.03–100.00)	0.07
Biopsy type	41.73 (0.00–100.00)	0.18
Methods for identifying *b* value	0.00 (0.00–100.00)	0.86

### Sensitivity analysis and publication bias

**Figure 9:**
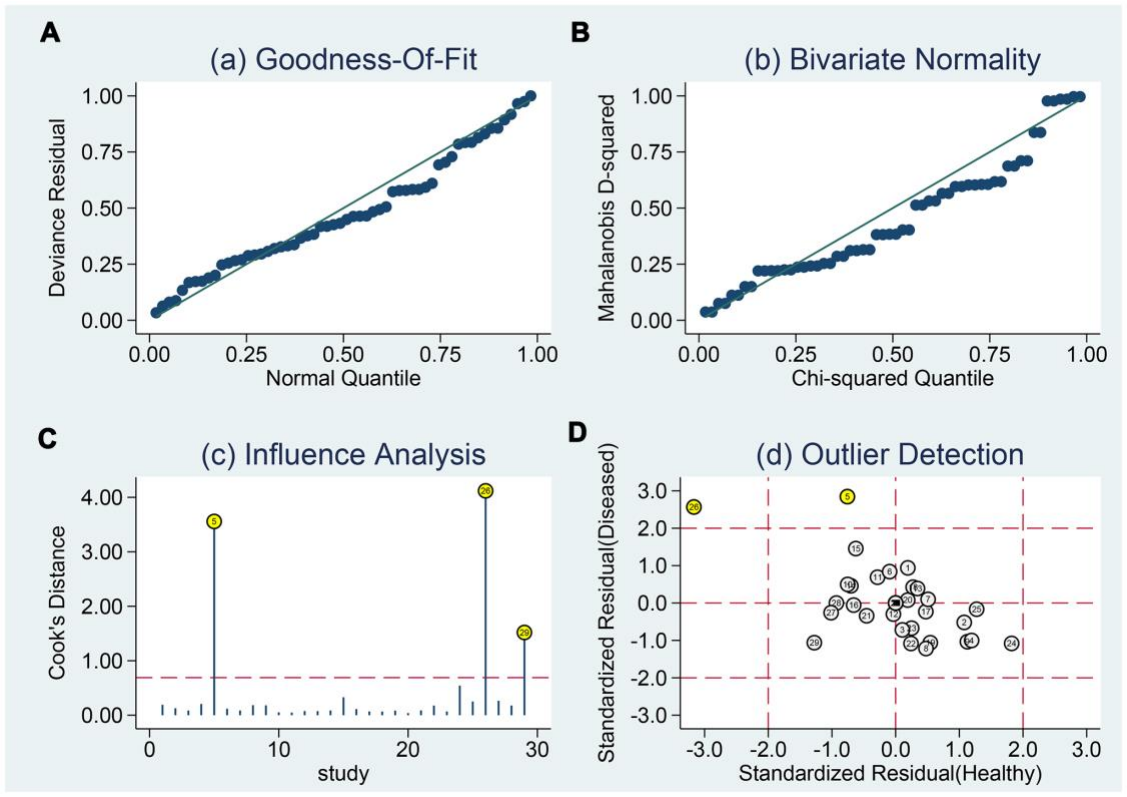
**Sensitivity analyses.** Graphical depiction of residual based goodness-of-fit (**A**), Bivariate normality (**B**), and influence (**C**) and outlier detection (**D**) analyses.

**Figure 10:**
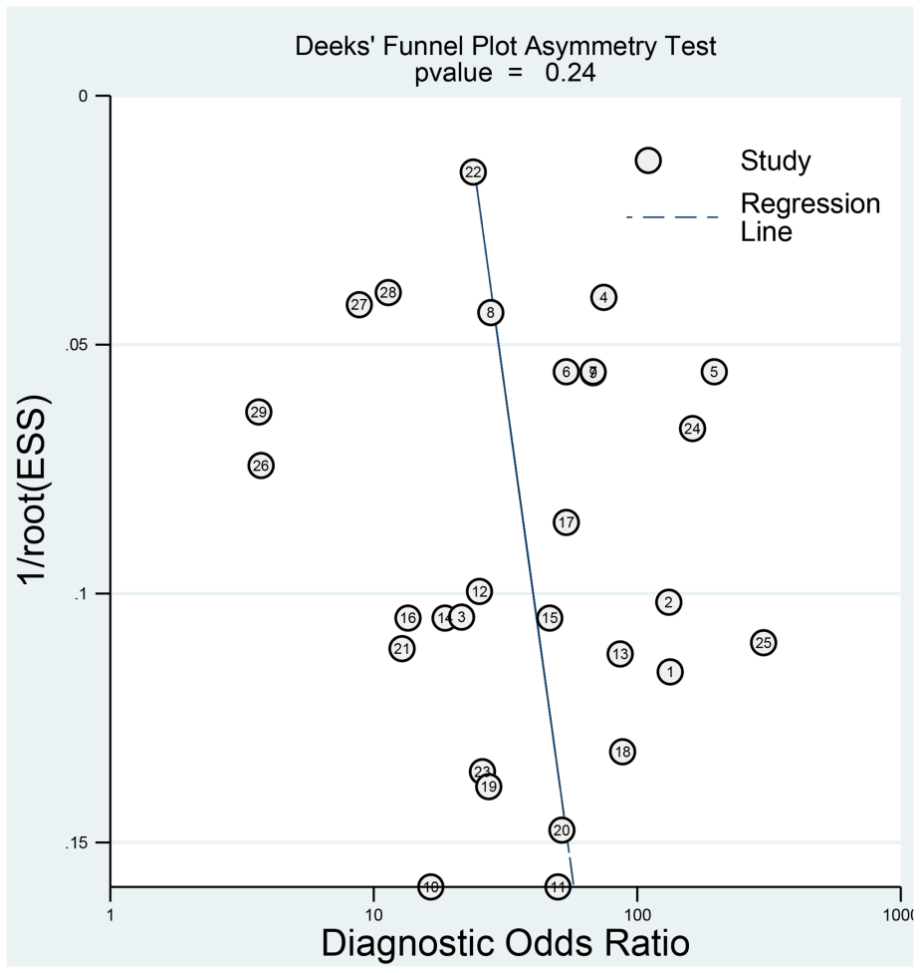
Deeks' funnel plot to evaluate the publication bias.

The sensitivity analysis is presented in Figure 9. The goodness of fit (A) and bivariate normality (B) show the degree of fitting of the regression line to the observed value. As shown, the observed value is distributed around the reference line. The observed values are stable. The influence analysis indicated that four studies may overestimate the pooled results. The outlier detection test indicated that two studies were out of the detection range. After excluding these studies, the pooled sensitivity, specificity did not change (results not show). In addition, we constructed Deek's plot, which indicated that there was no publication bias (t = −1.21, *p* = 0.240) (Figure 10).

## DISCUSSION

This meta-analysis compared twenty-four studies evaluating the use of high *b*-value DWI to diagnose prostate cancer. Importantly, the analysis indicated that high *b*-value DWI had a high diagnostic accuracy with a high sensitivity (0.85; 95% CI: 0.81–0.88), specificity (0.89; 95% CI: 0.86–0.92), and AUC (0.94; 95% CI: 0.91–0.96). Based on these results, high *b*-value DWI can be used to detect prostate cancer in clinical practice.

DWI is crucial for diagnosing prostate cancer when using MRI. Compared with central lesions, peripheral lesions are easier to assess using DWI, which is consistent with the Prostate Imaging Reporting and Data System version 2 scoring system. The human prostate is a highly heterogeneous organ at the cellular level, and structural tissues show changes during the early stages on a scale of micrometers or smaller for many prostate pathologies. The ability of DWI to detect prostate cancer relies on the shrinkage of glands, tight cell arrangement, and increased parenchyma density, such that water diffuses slower in prostate cancer tissues than in normal prostate tissues [33–35], [37–39]. The *b*-value, which needs to be selected carefully in clinical applications, is a key parameter reflecting the sensitivity of DWI for detecting diffusional movements. A high *b*-value can better distinguish cancerous tissues from normal tissues; however, it also has some disadvantages, such as reducing the image signal-to-noise ratio, which obscures cancerous tissues [36]. But it is reported that Siemens developed Readout Segmentation of Long Variable Echo-trains DWI technology that adopts multiple excitation segmental readout for acquisition and K space filling, which significantly shortens echo time, reduces echo interval and improves image quality on DWI. The special targeting uniformity technology can also obtain the best magnetic field uniformity, further improve the magnetic field uniformity of complex parts, so as to further improve the image quality of DWI. This technology has been confirmed in several tumors [37–39].

There is no accepted standard range of *b*-values for DWI that is optimal for diagnosing prostate cancer.

Our results suggest that a high *b*-value is a robust tool for prostate cancer diagnosis. The high AUC (0.94; 95% CI: 0.91–0.96) together with the high pooled sensitivity (0.85; 95% CI: 0.81–0.88) and specificity (0.89; 95% CI: 0.86–0.92) signified its very good diagnostic accuracy. The PLR and NLR were 8.0 (95% CI: 6.2–10.4) and 0.17 (95% CI: 0.13–0.21), respectively. The former indicates that the rate of diagnosing prostate cancer using high *b*-value DWI is 8.0 times higher in prostate cancer patients than in patients without prostate cancer. The latter suggests that the possibility of high *b*-value DWI diagnosis not detecting prostate cancer in individuals with prostate cancer is 17%. That means a high possibility of prostate cancer exclusion. Therefore, high *b*-value DWI showed better diagnostic accuracy. In the present study, *b* values of all studies included in the meta-analysis are 2000s/mm^2^ or more, and We will recommend *b* value ≥ 2000s/mm^2^ as optimal standard for diagnosing PCa.

Although our study was carefully conducted, some issues were inevitable. To demonstrate the source of heterogeneity, we performed several subgroup analyses. The subgroups assessed in our study were study design, number of patients, mean age of the patients, MRI field intensity, *b*-values, and DWI diagnostic measures. Regarding study design, the sensitivity of the prospective studies was superior to that of the retrospective studies, despite no obvious differences between the two designs. Furthermore, the DOR was higher for the prospective studies than the retrospective studies, and the AUC showed a significant difference between the two groups. We therefore concluded that the study design was a source of the heterogeneity observed. MRI field intensity showed the most striking results in that the PLR and DOR were almost twice as high for the studies using 3.0 T than for those using 1.5 T, and the AUC showed a significant difference between the two groups. The MRI field intensity contributed more to the heterogeneity among studies than did the study design. The sensitivity of studies involving high *b*-values (2,000 s/mm^2^) was slightly lower and the specificity was slightly higher than that of studies involving ultrahigh *b*-values (> 2,000 s/mm^2^), and the DOR and AUC between the two groups showed no significant differences. With respect to the number of patients, the studies with > 50 patients (compared with ≤ 50 patients) showed a higher sensitivity, PLR, DOR, and AUC. The mean ages of the patients showed the same results as those for the number of patients, but the difference was not significant.

We also performed subgroup analysis for qualitative and quantitative evaluation, and the studies based on ADC values (quantitative) versus visual evaluation (qualitative) showed no differences, suggesting that they had little or no contribution to the heterogeneity among studies. Although visual evaluation relies on the experience and skills of the performer, it results in no overall changes. Although the diagnostic accuracy was almost indifferent, there were still differences in imaging characteristics. A previous study found that the image deformation of DWI is smaller, the lesion contrast is higher in qualitative analysis, and the ADC value of DWI sequences shows better repeatability in quantitative analysis than standard DWI sequences [40]. The Prostate Imaging Reporting and Data System version 2.1 has recommended qualitative evaluation of DWI [41].

There were some limitations for this study. (1) The heterogeneity was high within studies. Even when we conducted subgroup analyses, the heterogeneity was carefully considered. The meta-regression indicated that the year of publication and population setting can affect the estimations. Besides, human prostate cancer cells are heterogenous, containing a variety of cancer cells with phenotypical and functional discrepancies, and this may generate heterogeneity. However, almost none of studies provided prostate cancer cell types, all studies just distinguish PCa from the tissues. Further research was required.

(2) The prostate cancer stage of the patients in the selected studies was not clear, which may have influenced the diagnostic accuracies. (3) The language of the searched studies was restricted to English and Chinese, which may have reduced the representativeness of the included studies. (4) It is needless to say that repeatability of DWI signal decay derived parameters needs to be evaluated because high repeatability of measurements is a prerequisite for quantitative patient tailored treatment planning and therapy monitoring. Previous studies found that Monoexponential model demonstrated the highest repeatability and clinical values in the regions - of interest-based analysis of prostate cancer DWI. However, included studies did not introduce used modeling and this study was based on the *b* values in the range of 0–500/mm^2^. Modeling evaluation based on high *b*-values are required [42].

In summary, high *b*-value DWI showed high diagnostic accuracy in the qualitative and quantitative evaluation of prostate cancer. We should consider the possibility of its clinical application, although studies with large sample sizes and higher quality are needed, particularly for quantitative evaluation. In addition, publication bias should be carefully considered when interpreting and applying our results.

## METHODS

This meta-analysis was performed and reported according to the Preferred Reporting Items for Systematic Reviews and Meta-Analyses (PRISMA) guidelines listed in the PRISMA statement [43]. The PRISMA statement is provided in Supplementary Material 5.

### Literature search

A comprehensive systematic literature search in the PubMed, Excerpta Medica Database (EMBASE), Cochrane Library, China National Knowledge Infrastructure, China Biology Medicine disc, and Wanfang databases was conducted to identify studies investigating the diagnostic performance of high *b*-value DWI for detecting prostate cancer. The search query combined synonyms and related terms of prostate cancer (“prostate disease,” “prostate tumor,” “prostate lesions,” and “PCa”), high *b*-value (“strong *b*-value,” “multiple *b*-value,” and “ultra-high *b*-value”), DWI, and diagnostic accuracy (“diagnostic performance,” “sensitivity,” “specificity,” and “receiver operator characteristic curve”). Logical operators (AND, NOT, and OR) were then used to conduct comprehensive combinations of these terms. The details of search strategy were provided in the Supplementary Material 6. Studies were restricted to English and Chinese languages, and the time span of the studies was January 1, 1995, to April 30, 2021. The references included in the identified papers were also screened to expand the range of our search.

### Selection criteria

Two reviewers (LC and LN) independently conducted the study selection. Controversies were settled by discussion. Only studies that met all of the following criteria were chosen: (1) a retrospective or prospective design was used; (2) the purpose of the study was to evaluate the diagnostic value of high *b*-value DWI in prostate cancer alone or data for assessing accuracy of high *b* value for prostate cancer can be extracted. (3) the study used *b*-values ≥ 2000 s/mm^2^; (4) the study included ≥ 30 patients; (5) histopathological results (as the gold standard) were available for all patients; and (6) sufficient information was provided to establish 2 × 2 contingency tables and to calculate the sensitivity and specificity for detecting prostate cancer. Studies were excluded if they satisfied any of the following criteria: (1) reviews, case reports, dissertations, or unpublished articles; (2) inclusion of animal experimental data; and (3) combination with other MRI modalities (T2-weighted or dynamic contrast-enhanced imaging) to evaluate the diagnostic performance of high *b*-value DWI for prostate cancer.

### Data extraction

One reviewer independently collected the data from the included studies using normative tables. The other reviewer double-checked this process. The following information was collected from the studies: author(s), country, study design, numbers of patients and lesions, mean age of the patients, PSA level range, MRI field intensity, MRI supplier, coil type, *b*-value, DWI diagnostic measures, population setting, biopsy type, diffusion times, DWI postprocessing, evaluation type (quantitively or qualitive), and methods for identifying the *b*-value. In addition, the numbers of TP (true positive), TN (true negative), FN (false negative) and FP (false positive) cases were collected to calculate the sensitivity and specificity. Disagreements in the data extraction findings were resolved via discussion or adjudication with a third reviewer.

### Quality evaluation

Each paper’s quality was assessed using the Quality Assessment of Diagnostic Accuracy Studies 2, a validated tool specifically designed to evaluate diagnostic accuracy studies via four domains: flow and timing, patient selection, index test, and reference standard. A risk of bias existed in all four domains, but applicability concerns existed in only the last three domains. Both risk of bias and applicability concerns were graded as low, unclear, or high [44]. This step was conducted independently by two reviewers, and controversies were settled by discussion or by consulting a third party.

### Statistical analysis

Using uses an exact binomial rendition of the bivariate mixed-effects regression model developed by von Houwelingen for treatment trial meta-analysis and modified for synthesis of diagnostic test data [45]. The pooled sensitivity, specificity, positive likelihood ratio (PLR), negative likelihood ratio (NLR), diagnostic odds ratio (DOR), and area under the SROC curve (AUC) along with 95% confidence intervals (CIs) were determined using Stata 14.0 software (https://www.stata.com/). The heterogeneity among studies was quantified using the *Q* test and I^2^ statistic [46]. The *Q* test was defined by Cochran and is calculated by summing the squared deviations of each study’s effect estimate from the overall effect estimate, weighting the contribution of each study by its inverse variance. The I^2^ index measures the extent of true heterogeneity, dividing the difference between the result of the *Q* test and its degrees of freedom by the *Q* value itself and multiplying by 100 [43]. An I^2^ > 50% and *P* < 0.05 were considered to indicate heterogeneity. Subgroup analysis was used to evaluate the heterogeneity among groups. The study design, number of patients, mean age of the patients, MRI field intensity, DWI diagnostic measures, and *b*-value were compared by subgroup analyses. The aim of our study was to understand the effect of high *b*-values and standard *b*-values on the diagnostic accuracy of prostate cancer, but without further analyses of the effect between high *b*-values and ultrahigh *b*-values, subgroup analyses were conducted. The concrete comparisons were (1) study design: prospective versus retrospective; (2) number of patients: ≤ 50 versus > 50; (3) mean age: ≤ 65 years versus > 65 years; (4) MRI field intensity: 1.5 T versus 3 T; (5) *b*-value: high (2,000) versus ultrahigh (> 2,000); and (6) DWI diagnostic measure: ADC values versus visual evaluations. Fagan’s nomogram was used to show the relevance of the prior test probability, likelihood ratio, and posterior test probability. Publication bias was visualized using Deek’s funnel plot. Meta-regression was performed for exploring the heterogeneity within studies. All statistical computations were conducted using Stata 14.0 software (Stata Corp, College Station, TX, USA), and the results were considered significant at *P* < 0.05.

### Data availability

All data was within the manuscript without any restriction.

## Supplementary Materials

Supplementary Materials 2-4

Supplementary Material 1

Supplementary Material 5

Supplementary Material 6
