# Supplementary material for: Diagnostic accuracy of high b-value diffusion weighted imaging for patients with prostate cancer: a diagnostic comprehensive analysis
Source: Aging (Albany NY). 2021 Jun 22;13(12):16404–24. doi: 10.18632/aging.203164 (PMC8266335; doi:10.18632/aging.203164)
Supplement: Supplementary Materials 2-4 [file aging-13-203164-s001.pdf]

## SUPPLEMENTARY MATERIALS

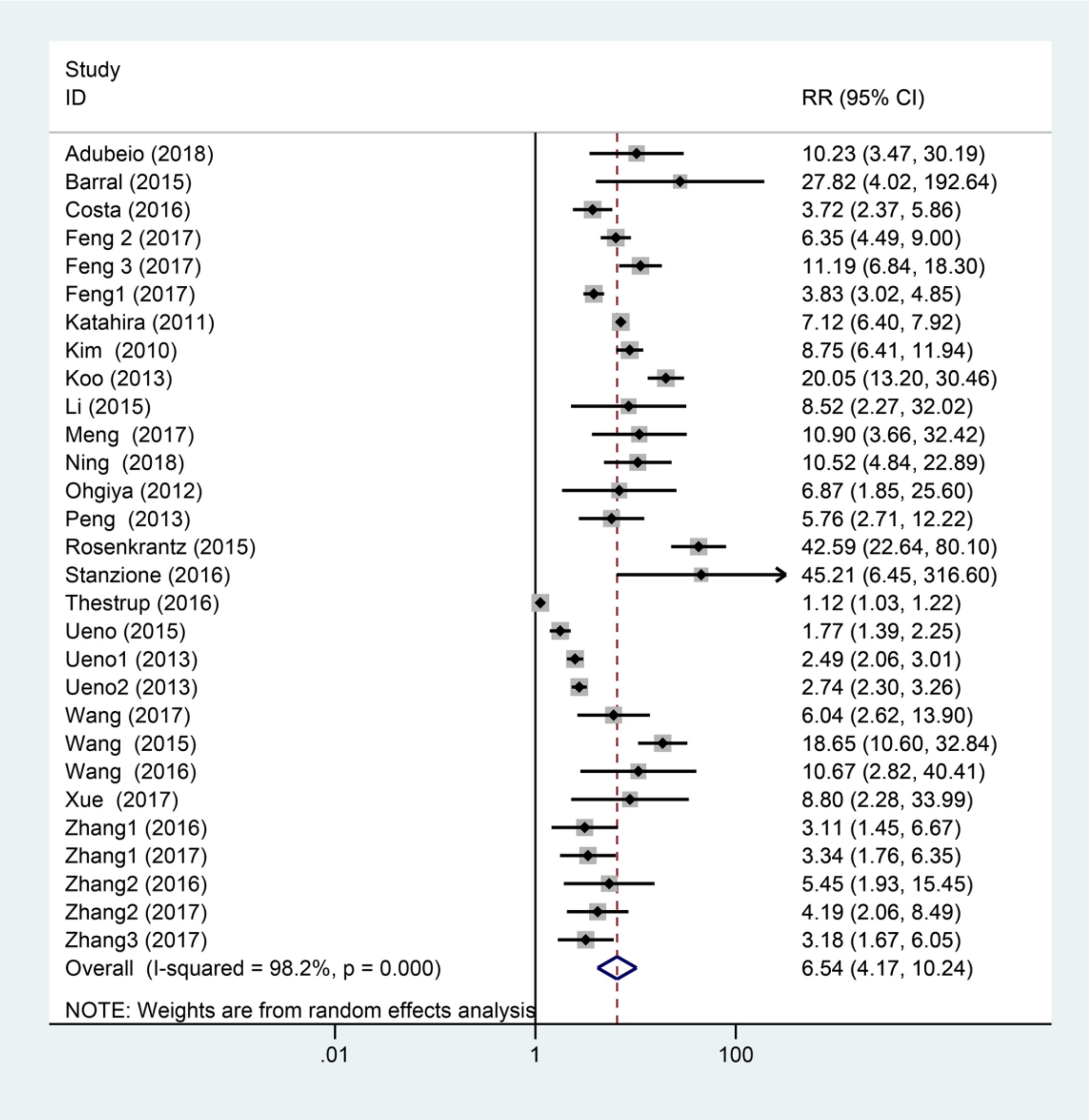


**Supplementary Material 2. Forest plot of pooled positive likelihood ratio of diagnostic accuracy of high *b*-value DWI for detecting prostate cancer.**

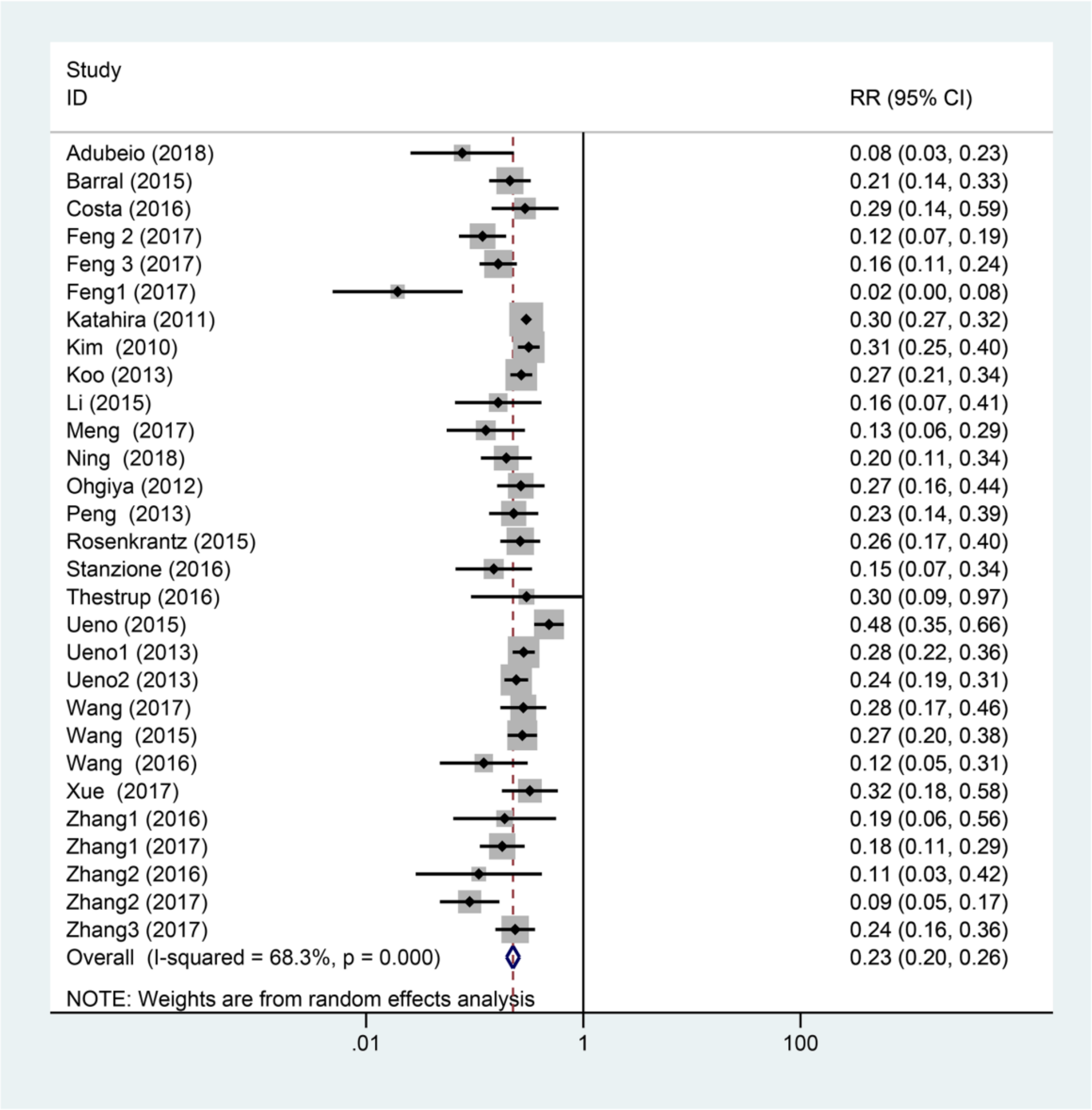


**Supplementary Material 3. Forest plot of pooled negative likelihood ratio of diagnostic accuracy of high b-value DWI for detecting prostate cancer.**

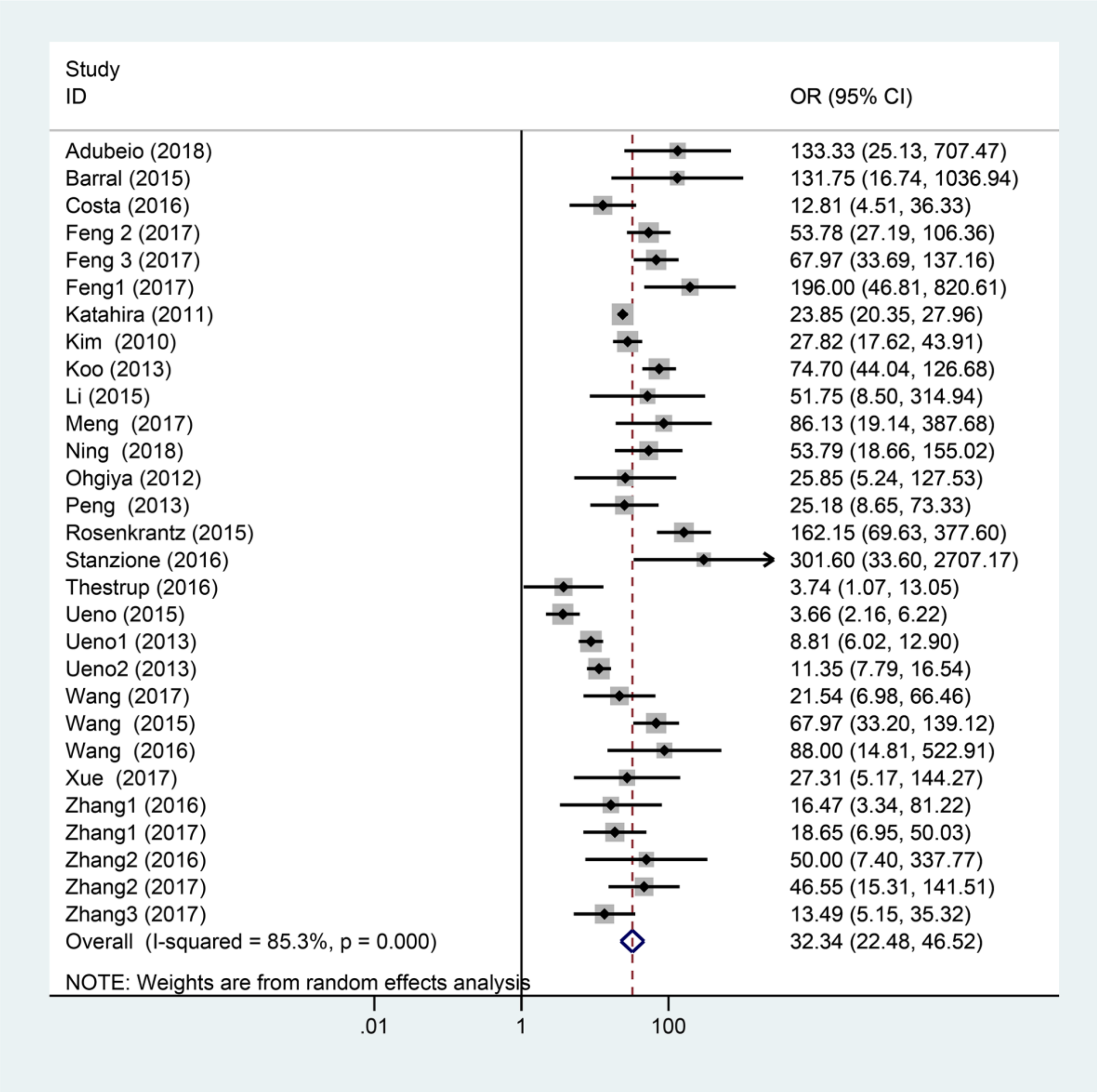


**Supplementary Material 4. Forest plot of pooled diagnostic odds ratio of diagnostic accuracy of high *b*-value DWI for detecting prostate cancer.**
