# Supplementary material for: Diagnostic accuracy of high b-value diffusion weighted imaging for patients with prostate cancer: a diagnostic comprehensive analysis
Source: Aging (Albany NY). 2021 Jun 22;13(12):16404–24. doi: 10.18632/aging.203164 (PMC8266335; doi:10.18632/aging.203164)
Supplement: Supplementary Material 1 [file aging-13-203164-s002.doc]

**Supplementary Material 1.** List of excluded references and reasons for exclusion.

| NO. | References | Reason |
| --- | --- | --- |
| 1 | Li L, Wang A, Wang J, Wang Y, Y J. Value of high-b-value Diffusion-weighted Imaging in the Differential Diagnosis of Prostate Cancer and Prostatitis. Chin J Med Imaging.（2012）12: 887-889. | Sample size<30 |
| 2 | Colvin SD, Cason DE, Galgano SJ, Triche BL, Gordetsky J, Rais BS, et al. Fusion of high B-value diffusion-weighted and T2-weighted MR images increases sensitivity for identification of extraprostatic disease in prostate cancer. Clin Imag. (2020) 68. | bpMRI |
| 3 | Li X. Application value of high B value DWI scan combined with dynamic contrast-enhanced MRI in diagnosis of prostate cancer. China Modern Pharmacy. (2019) 13: 61-62. | bpMRI |
| 4 | Zhou J, Meng Y, Xu M. Application of high B value diffusion weighted imaging in the diagnosis of prostate cancer. J Med Theor&Prac. (2016) 29: 2974-2976. | Sample size<30 |
| 5 | Tom S, Keith G, Paul M. Inter-observer agreement of prostate cancer in high b-value diffusion weighted imaging with small and large field-of-view. Clin Radiol. (2016) 71. | Unrelated topic |
| 6 | Agarwal HK, Mertan FV, Sankineni S, Bernardo M, Senegas J, Keupp J, et al. Optimal high b-value for diffusion weighted MRI in diagnosing high risk prostate cancers in the peripheral zone. Journal of magnetic resonance imaging : JMRI. (2017) 45. | Non high b value |
| 7 | Andrew BR, Nainesh P, Andrea SK, Max XK, James SB, Samir ST, et al. Prostate Cancer Detection Using Computed Very High b-value Diffusion-weighted Imaging: How High Should We Go? Acad Radiol. (2016) 23. | Review |
| 8 | Rosenkrantz AB, Parikh N, Kierans AS, Kong MX, Babb JS, Taneja SS, et al. Prostate Cancer Detection Using Computed Very High b-value Diffusion-weighted Imaging: How High Should We Go? Acad Radiol. (2016) 23. | Duplicates |
| 9 | Rosenkrantz AB, Mannelli L, Kong X, Niver BE, Berkman DS, Babb JS, et al. Prostate cancer: utility of fusion of T2-weighted and high b-value diffusion-weighted images for peripheral zone tumor detection and localization. Journal of magnetic resonance imaging : JMRI. (2011) 34. | Review |
| 10 | Liu J, Zhang H. High b -values of diffusion -weighted MR Imaging in diagnosis of prostate  cancerous and non-cancerous regions by GE3.0T. China Modern Med. (2011) 18: 9-10. | No data for analysis |
| 11 | S. Jendoubi，M. Wagner，S. Montagne，et al. MRI in the diagnosis of prostate cancer: whether the calculated high B value DWI can replace the self-collected DWI. Inter J Med Radiology,2019,42(06):749-50. | Abstract |
| 12 | Roshan AK, Joshua K, Tyler MS, et al. Relationship between kurtosis and bi-exponential characterization of high b-value diffusion-weighted imaging: application to prostate cancer. Acta Radiologica 2018; 59. | No useful data |
| 13 | Boesen L, Norgaard N, Logager V, Balslev I, Bisbjerg R, Thestrup KC, et al. Assessment of the Diagnostic Accuracy of Biparametric Magnetic Resonance Imaging for Prostate Cancer in Biopsy-Naive Men: The Biparametric MRI for Detection of Prostate Cancer (BIDOC) Study. JAMA Netw Open. (2018) 1: e180219. doi:10.1001/jamanetworkopen.2018.0219 | biparametric |
| 14 | Boesen L, Norgaard N, Logager V, Balslev I, Bisbjerg R, Thestrup KC, et al. Prebiopsy Biparametric Magnetic Resonance Imaging Combined with Prostate-specific Antigen Density in Detecting and Ruling out Gleason 7-10 Prostate Cancer in Biopsy-naive Men. Eur Urol Oncol. (2019) 2: 311-319. doi:10.1016/j.euo.2018.09.001 | Duplicates |
| 15 | Rais-Bahrami S, Siddiqui MM, Vourganti S, Turkbey B, Rastinehad AR, Stamatakis L, et al. Diagnostic value of biparametric magnetic resonance imaging (MRI) as an adjunct to prostate-specific antigen (PSA)-based detection of prostate cancer in men without prior biopsies. Bju Int. (2015) 115: 381-388. doi:10.1111/bju.12639 | biparametric |
| 16 | Y. Zhang，S. A. Wells，B. L. Triche. Stimulated -echo diffusion -weighted imaging with moderate b values for the detection of prostate cancer. Inter J Med Radiology,2020,43(04):501. | Abstract |
| 17 | Jie Ceng，Longlin Yin，Ju Sun. Study on the Application Value of IVIM Imaging with Different b Value Combinations in the Diagnosis of Prostate Cancer. J Clin Radiology. 2020,39(07):1367-1372.(in Chinese) | Non-high b value |
| 18 | Xiaoxue Ye，Chongyong Xu，Yongfei Zhou. Study on the Application Value of IVIM Imaging with Different b Value Combinations in the Diagnosis of Prostate Cancer.,2020,27(14):1671-1674.(in Chinese) | Duplicates |
| 19 | Jambor I, Bostrom PJ, Taimen P, Syvanen K, Kahkonen E, Kallajoki M, et al. Novel biparametric MRI and targeted biopsy improves risk stratification in men with a clinical suspicion of prostate cancer (IMPROD Trial). J Magn Reson Imaging. (2017) 46: 1089-1095. doi:10.1002/jmri.25641 | biparametric |
| 20 | Hamed K, Ravi TS, Elisabeth W, Berthold K, Marcel DN, Thitinan C, et al. Image quality and diagnostic accuracy of complex-averaged high b value images in diffusion-weighted MRI of prostate cancer. Abdom Radiol. (2019) 44. | duplicates |
| 21 | Low-to-high b value DWI ratio approaches in multiparametric MRI of the prostate: feasibility, optimal combination of b values, and comparison with ADC maps for the visual presentation of prostate cancer. Quant Imag Med Surg. (2018) 8. | No data for extraction |
| 22 | Ma S, Xu K, Xie H, Wang H, Wang R, Zhang X, et al. Diagnostic efficacy of b value (2000 s/mm2) diffusion-weighted imaging for prostate cancer: Comparison of a reduced field of view sequence and a conventional technique. Eur J Radiol. (2018) 107. | Duplicates |
| 23 | Shen J, Xue F, Cai Y, Lu JG, Wang J. The clinical value of ultra-high b value diffusion weighted magnetic resonance imaging combined with ultrasound elastography for the diagnosis of prostate cancer. Imaging Res Med Application. (2018) 2: 96-98. | Multiparameters |
| 24 | Yang C, Yang Q, Dang S, Duan HF, Ma G, Duan X. Effect of Two Different Diffusion Weighted Imaging with Ultra-high b Values on Image Quality in Prostate Cancer MRI. Chin J Med Imaging. (2018) 26: 606-611. | No data for extraction |
| 25 | Wang W, Shao ZH, Zeng JQ, Wu DL, Wang GL, Wang PJ. [Effects of different computed high b-values on diffusion weighted imaging scores in Prostate Imaging Reporting and Data System version 2 of prostate cancer in peripheral zone]. Zhonghua yi xue za zhi. (2017) 97. | Duplicates |
| 26 | Grant KB, Agarwal HK, Shih JH, Bernardo M, Pang Y, Daar D, et al. Comparison of calculated and acquired high b value diffusion-weighted imaging in prostate cancer. Abdom Imaging. (2015) 40. | No data for extraction |
| 27 | Kinzya BG, Harsh KA, Joanna HS, Marcelino B, Yuxi P, Dagane D, et al. Comparison of calculated and acquired high b value diffusion-weighted imaging in prostate cancer. Abdom Imaging. (2015) 40. | No data for extraction |
| 28 | T., Metens, D., Miranda, J., Absil C., Matos What is the Optimal B Value in Diffusion-Weighted MR Imaging to Depict Prostate Cancer at 3T? Eur. Radiol. 22, (2012). | No data |
| 29 | Park SY, Kim CK, Park BK, Kwon GY. Comparison of apparent diffusion coefficient calculation between two-point and multipoint B value analyses in prostate cancer and benign prostate tissue at 3 T: preliminary experience. AJR. American journal of roentgenology. (2014) 203. | Non-high b value |
| 30 | Ueno Y, Takahashi S, Ohno Y, Kitajima K, Yui M, Kassai Y, et al. Computed diffusion-weighted MRI for prostate cancer detection: the influence of the combinations of b-values. The British journal of radiology. (2015) 88. | Combinations of b-values |
| 31 | Jin TK, Sheng X, Bradford JW, Baris T, Peter LC, Peter AP, et al. Automated prostate cancer detection using T2a weighted and high b value diffusiona weighted magnetic resonance imaging. Med Phys. (2015) 42. | No data |
| 32 | de Perrot T, Scheffler M, Boto J, Delattre BMA, Combescure C, Pusztaszeri M, et al. Diffusion in prostate cancer detection on a 3T scanner: How many b-values are needed? Journal of magnetic resonance imaging : JMRI. (2016) 44. | Review |
| 33 | Vallini V, Ortori S, Boraschi P, Manassero F, Gabelloni M, Faggioni L, et al. Staging of pelvic lymph nodes in patients with prostate cancer: Usefulness of multiple b value SE-EPI diffusion-weighted imaging on a 3.0 T MR system. European journal of radiology open. (2016) 3. | Unrelated topic |
| 34 | Zhang Y, Wu C, Bao M, Li H, Yan X, Liu X, et al. New RESOLVE-Based Diffusional Kurtosis Imaging in MRI-Visible Prostate Cancer: Effect of Reduced b Value on Image Quality and Diagnostic Effectiveness. AJR. American journal of roentgenology. (2016) 207. | Unrelated topic |
| 35 | Ayumu K, Tsutomu T, Teruki S, Naoki K, Yoshiyuki M, Akira Y, et al. Incremental value of high b value diffusion-weighted magnetic resonance imaging at 3-T for prediction of extracapsular extension in patients with prostate cancer: preliminary experience. La radiologia medica. (2017) 122. | No data |
| 36 | Zeng J, Deng, Q, Li S, Chen S. Diagnostic value of DWI with ultrahigh b-value combined with DCE-MRI in prostate cancer. China Med,53(8):911-914 | Combined methods |
| 37 | Mostafa A, Jean PS, Kaela G, Lucy S, Ali J, Hang Y, et al. Biparametric vs multiparametric prostate magnetic resonance imaging for the detection of prostate cancer in treatment‐naive patients: a diagnostic test accuracy systematic review and meta‐analysis. Bju Int. (2019) 124. | Review |
