# Supplementary material for: Diagnostic accuracy of high b-value diffusion weighted imaging for patients with prostate cancer: a diagnostic comprehensive analysis
Source: Aging (Albany NY). 2021 Jun 22;13(12):16404–24. doi: 10.18632/aging.203164 (PMC8266335; doi:10.18632/aging.203164)

**Supplementary Material 6.** Search strategy and Search terms.

**1. PubMed:**

(((((prostate cancer [MeSH Terms])) OR (prostate disease [MeSH Terms])) OR (prostate tumor [MeSH Terms])) OR (prostate lesions [MeSH Terms])) OR (PCa) ) AND ((((((((DWI[Title])) OR (diffusion weighted imaging[Title])) OR (MRI[Title])) OR (dynamic contrast enhanced imaging[Title])) OR (diffusion weighted MR imaging [Title])) OR (MR imaging [Title])) OR (diffusion weighted )) OR (ultra-high b value)

**2. Web of Science:**

Step 1: prostate cancer (Topic) or prostate disease (Topic) or prostate tumor (Topic) or prostate lesions (Topic) or PCa (Topic) Databases= WOS, BCI, KJD, MEDLINE, RSCI, SCIELO Timespan=All years

Search language=Auto

Step 2: DWI (Topic) or diffusion weighted imaging (Topic) or MRI (Topic) or dynamic contrast enhanced imaging (Topic) or diffusion weighted MR imaging (Topic) or diffusion weighted (Topic) or ultra-high b value (Topic)

Databases= WOS, BCI, KJD, MEDLINE, RSCI, SCIELO Timespan=All years

Search language=Auto

Step 3: #1 AND #2

Databases= WOS, BCI, KJD, MEDLINE, RSCI, SCIELO Timespan=All years

Search language=Auto

**3. Embase:**

Step 1: 'prostate cancer':ti,ab,kw OR 'prostate disease':ti,ab,kw OR 'prostate tumor':ti,ab,kw OR 'prostate lesions':ti,ab,kw OR pca:ti,ab,kw

Step 2: dwi:ti,ab,kw OR 'diffusion weighted imaging':ti,ab,kw OR mri:ti,ab,kw OR 'dynamic contrast enhanced imaging':ti,ab,kw OR 'diffusion weighted mr imaging':ti,ab,kw OR 'diffusion weighted':ti,ab,kw OR 'ultra-high b value':ti,ab,kw

Step 3: #1 AND #2

**4.CNKI, China Biology Medicine disc and Wanfang:**


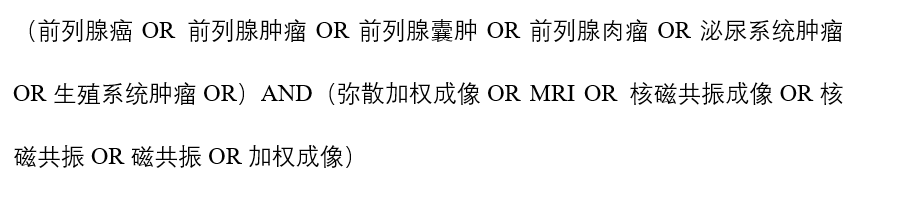

Supplement: Supplementary Material 6 [file aging-13-203164-s004.docx]
